# Enhancing forecast accuracy using combination methods for the hierarchical time series approach

**DOI:** 10.1371/journal.pone.0287897

**Published:** 2023-07-17

**Authors:** Rania A. H. Mohamed

**Affiliations:** Department of Statistics, Mathematics, and Insurance, Faculty of Commerce, Port Said University, Port Fouad, Port Said, Egypt; King Fahd University of Petroleum and Minerals, SAUDI ARABIA

## Abstract

This study aims to investigate whether combining forecasts generated from different models can improve forecast accuracy rather than individual models using the hierarchical time series. Various approaches of hierarchical forecasting have been considered; a bottom-up, top-down, and an optimal combination approach. Autoregressive moving averages (ARIMA) and exponential smoothing (ETS) were used as forecasting models in creating forecasting for all levels in the hierarchy to show the effect of different forecasting methods for each hierarchical model. The results indicated that the Minimum Trace Sample estimator (MinT-Sample) and the bottom-up approaches with the ARIMA model have good predictive performance than other approaches. Moreover, the forecasts from the MinT-Sample and bottom-up approaches were combined using five different combining methods. The experimental results showed that the (AC) method is superior to all other combining methods and more accurate than other individual models at level zero (international total trade in Egypt) and level one (total exports, and total imports). So, combining forecasts generated from different models by hierarchical time series leads to more accurate forecasting of the value of imports and exports which will improve the overall international trade performance, and that is through using the forecasting values of imports and exports to plan for improving the trade balance and drawing up a more efficient production policy. Finally, the study recommends using hierarchical forecasting methods in the areas of international trade, and the Ministry of Commerce and Industry could adopt the results of this study to produce precise forecasts for international trade. Moreover, the results of this study are to be a guide for the researchers to apply these approaches in other fields to improve the performance of forecasting.

## 1 Introduction

Hierarchical time series, are multiple time series that are organized hierarchically and can be grouped at many different levels into groups based on geographic location, products, or other features. There are many specialized strategies, such as bottom-up, top-down, or a combination of the two, called “middle-out” and the optimal combination approach, [1]. In [2], Hyndman et al. propose an approach to hierarchical forecasting which provides optimal forecasts that are better than forecasts produced by either a top-down or a bottom-up approach. The method is based on independently forecasting all series at all levels of the hierarchy and then using a regression model to optimally combine and reconcile these forecasts. The resulting revised forecasts add up appropriately across the hierarchy, are unbiased, and have minimum variance amongst all combination forecasts under some simple assumptions. The simulation study shows that the method performs well compared to the top-down approach and the bottom-up method, the proposed method demonstrates by forecasting Australian tourism demand where the data are disaggregated by the purpose of travel and geographical region. In [3], Makoni and Chikobvu presented a paper that aims to model and forecast the Victoria Falls Rainforest tourism demand using hierarchical forecasting methods, the top-down, bottom-up, and optimal combination approaches are adopted. The exponential smoothing techniques (EST) and the autoregressive integrated moving average (ARIMA) methods are the forecasting methods considered. Accuracy measures indicated the bottom-up approach under ARIMA models as the best approach to the data and produced sensible future tourism forecasts. Oliveira and Ramos [4] investigate the relative performance of independent and reconciled forecasting approaches, using real data from a Portuguese retailer, two alternative forecasting model families for generating the base forecasts are considered; namely, state space models and ARIMA. Appropriate models from both families are chosen for each time series by minimizing the bias-corrected Akaike information criteria. The results show significant improvements in forecast accuracy, providing valuable information to support management decisions. And it is clear that reconciled forecasts using the Minimum Trace Shrinkage estimator (MinT-Shrink) generally improve on the accuracy of the ARIMA base forecasts for all levels and for the complete hierarchy, across all forecast horizons. Rehman and et al. [5], show that hierarchical time series arise in manufacturing and service industries when the products or services have a hierarchical structure, and top-down and bottom-up methods are commonly used to forecast the hierarchical time series, one of the critical factors that affect the performance of the two methods is the correlation between the data series, which this study attempts to resolve this problem and shows that the top-down method performs better when data have a high positive correlation compared to a high negative correlation and a combination of forecasting methods may be the best solution when there is no evidence of the correlation ship. The results show that the regression-based, VAR-COV, and Rank-based methods perform better compared to the other methods. Silveira and Azevedo [6], analyzed hourly power generation in Brazil (2018–2020), grouped according to each of the electrical subsystems and their respective sources of generating energy. The objective was to calculate the accuracy of the main measures of aggregating and disaggregating the forecasts of the Autoregressive Integrated Moving Average and Error, Trend, and Seasonal models (ETS). Specifically, the hierarchical approaches were analyzed: bottom-up, top-down, and optimal reconciliation. The optimal reconciliation models showed the best mean performance, considering the primary predictive windows. It was also found that energy forecasts in the South subsystem presented greater inaccuracy compared to the others, which signals the need for individualized models for this subsystem. Makoni and et al. [7], presented a paper whose objectives are as follows: First, to adopt the hierarchical forecasting methods in modeling and forecasting international tourist arrivals in Zimbabwe; Second, to come up with Zimbabwe international tourist arrivals Prediction Intervals (PIs) in Quantile Regression Averaging (QRA) to hierarchical tourism forecasts. Zimbabwe’s monthly international tourist arrivals data from January 2002 to December 2018 was used. The data set was disaggregated according to the purpose of the visit. Three hierarchical forecasting approaches, top-down, bottom-up, and optimal combination approaches were applied to the data. The results showed the superiority of the bottom-up approach over both the top-down and optimal combination approaches. Forecasts indicate a general increase in aggregate series. The combined methods provide new insight into modeling tourist arrivals.

This study presents a new main motivation for conducting to investigate whether combining forecasts generated from different hierarchical time series models can improve forecast accuracy rather than using individual models.

In summary, the contributions to the study are:

1. Using hierarchical time series through three different approaches; the bottom-up approach, the top-down approach, and the optimal combination approach
2. Combining the forecasts from the best hierarchical forecasting approaches using five combination methods, the Simple Average method, Geometric Mean, Variance—Covariance method, AKAIKE Weights, and AC method.
3. Comparing the performance of the individual forecasts to the combination forecasts using Mean Absolute Percentage Error (MAPE), and Root Means Error (RMSE) as the most popular forecast error measures.
4. Providing new insight into modeling international trade in Egypt that benefits the government, exporters, and importers.

This study is organized as follows. Section 2 provided a brief description of the methodology. The data is provided in Section 3, and the results are presented in Section 4. Finally, the conclusions are summarized.

## 2 Methodology

There are many applications in the field of business and economics that are organized hierarchically and can be grouped at several different levels into groups based on geography, products, or some other features, which are called hierarchical time series such as international trade data. There are common approaches are using for forecasting hierarchical time series; bottom-up, top-down, and Optimal combination approach proposed by Hyndman et al. [2] which has many advantages; Presents point forecasts that are reconciled across the levels of the hierarchy; allows for the interactions and correlations between the series at every level, Presents estimates of forecast uncertainty which are reconciled across the levels; the approach is flexible and provides optimal forecasts under some simple assumptions. The hierarchical forecasting approaches can capture the changes in international trade data and generate accurate forecasts. This section is divided into three parts, the first one briefly introduced the hierarchical approaches used in this study, the second part introduced the forecasting models (ARIMA and ETS) and the third part presented some combining methods.

### 2.1 Hierarchical forecasting approaches

Three approaches are used in this study: bottom-up, top-down, and optimal combination.

#### 2.1.1 The bottom-up approach

One of the most common approaches used for hierarchical forecasting is the bottom-up approach, which requires first providing forecasts for each series at the bottom level, and then aggregating them to provide forecasts for all the levels of the hierarchal structure. The advantage of this approach is that, by modeling the data at the most disaggregated bottom level, no information is lost due to aggregation. Therefore, the dynamics of the individual series can be better captured. However, bottom-level data can be quite noisy, and, therefore, more challenging to model.

The hierarchical methods can be represented by the general form:

$$\begin{array}{c} {\tilde{Y}}_{n}\left(h\right)=SP{\hat{Y}}_{n}\left(h\right) \end{array}$$ (1)

Where *S* is the *m* × *m*_*k*_ summing matrix, and *P* is a Matrix of order *m*_*k*_ × *m*, the role of *P* changes depending on the hierarchical approach. To represent the bottom-up approach using Eq (1),

$$\begin{array}{c} P=[0_{mk\times (m-mk)}|I_{mk}] \end{array}$$

where 0_*i*×*j*_ is the *i* × *j* null matrix, the role of *P* is to extract the bottom-level forecasts, which are subsequently aggregated by the summation matrix *S* to provide the revised forecasts for the whole hierarchy. For more detail, refer to [6, 8].

#### 2.1.2 Top-down approach

The top-down approach is the other common approach in hierarchical forecasting; the approach disaggregates the forecasts of the total series and distributes these down the hierarchy depending on historical data proportions, to represent the top-down approach using Eq (1),

$$\begin{array}{c} P=[p|0_{mk\times (m-1)}], \end{array}$$

Where *p* = [*p*1, *p*2, …, *p*_*mk*_]′ is a set of proportions for the bottom-level series, the role of P is to distribute the top-level forecasts to forecasts for the bottom-level series. Different top-down forecasting methods lead to other proportionality vectors p.

In this study, three models of this approach are used [8].

1. Top-down forecasts based on the average historical proportions (Gross-Sohl method A) (TDGSA)
  $$\begin{array}{c} p_{j}=\frac{\sum_{t=1}^{n}\frac{Y_{j,t}}{Y_{t}}}{n}\;,\;j=1,\ldots ,m_{k} \end{array}$$
  Each proportion *p*_*j*_ reflects the average of the historical proportions of the bottom level series *Y*_*j*,*t*_ over the period t = 1, …, n, relative to the total aggregate *Y*_*t*_
2. Top-down forecasts based on the proportion of historical averages (Gross-Sohl method F) (TDGSF)
  $$p_{j}=\frac{\sum_{t=1}^{n}\frac{Y_{j,t}}{n}}{\sum_{t=1}^{n}\frac{Y_{t}}{n}}\;,\;j=1,\ldots ,m_{k}$$
  Each proportion *p*_*j*_ captures the average historical value of the bottom level series *Y*_*j*,*t*_ relative to the average value of the total aggregate *Y*_*t*_.
3. Top-down forecasts using forecast proportions (TDFP)
  $$p_{j}=\prod_{\ell =0}^{k-1}\frac{{\hat{Y}}_{j,n}^{\left(\ell \right)}\left(h\right)}{{\hat{S}}_{j,n}^{\left(\ell +1\right)}\left(h\right)}\;,\;j=1,2,\ldots ,m_{k}$$
  where ${\hat{Y}}_{j,n}^{\left(\ell \right)}\left(h\right)$ is the h-step-ahead forecast and ${\hat{S}}_{j,n}\left(h\right)$ is the sum of the h-step-ahead forecasts below node *j* which are directly connected to node *j*. For more detail, refer to [6, 8, 9].

#### 2.1.3 Optimal combination approach

Hyndman et al. [2] suggested a new approach to forecasting hierarchical models; the method is based on independently forecasting all series at all levels of the hierarchy and then combining and reconciling these forecasts using a regression model. The base forecasts can be written as follows:

$$\begin{array}{c} {\hat{Y}}_{n}\left(h\right)=S\beta_{n}\left(h\right)+\varepsilon_{h} \end{array}$$ (2)

Where *β*_*n*_(*h*) = *E*[*Y*_*K*,*n*+*h*_ ∣ *Y*_1_, …, *Y*_*n*_] is the unknown mean of the bottom level *K*, *ε*_*h*_ has zero mean, and covariance matrix Var(*ε*_*h*_) = Σ_*h*_, then estimate *β*_*n*_(*h*) by treating Eq (2) as a regression equation and obtain forecasts for all levels of the hierarchy. If Σ_*h*_ was known, generalized least squares estimation is used to get the minimum variance unbiased estimate of *β*_*n*_(*h*) as:

$${\hat{\beta }}_{n}\left(h\right)=(S^{\prime }\Sigma_{h}^{+}S{)}^{-1}S^{\prime }\Sigma_{h}^{+}{\hat{Y}}_{n}\left(h\right)$$

Where $\Sigma_{h}^{+}$ is the Moore-Penrose generalized inverse of Σ_*h*_, the revised forecasts is given by:

$$\begin{array}{c} {\tilde{Y}}_{n}\left(h\right)=S{\hat{\beta }}_{n}\left(h\right)=SP{\hat{Y}}_{n}\left(h\right) \end{array}$$ (3)

Where $P=(S^{\prime }\Sigma_{h}^{+}S{)}^{-1}S^{\prime }\Sigma_{h}^{+}$; which satisfies the unbiasedness property *SPS* = *S*. This condition is valid for the bottom-up approach, although not for the top-down approach because of *SPS* ≠ *S*. So, the top-down approaches will never give unbiased forecasts, even if the base forecasts are unbiased. The variance of these forecasts is given by:

$$\text{Var}[{\tilde{Y}}_{n}\left(h\right)]=S{\left(S^{\prime }\Sigma_{h}^{+}S\right)}^{-1}S^{\prime }$$

In general, Σ_*h*_ is not known and is not identifiable [10]. The residuals from the regression model in Eq (2) are given by

$$\begin{array}{cc} & {\tilde{\varepsilon }}_{h}={\hat{Y}}_{n}\left(h\right)-{\tilde{Y}}_{n}\left(h\right)=(I_{m}-SP){\hat{Y}}_{n}\left(h\right)\\ & Var({\tilde{\varepsilon }}_{h}|I_{n})=(I_{m}-SP)\Sigma_{h}{\left(I_{m}-SP\right)}^{\prime } \end{array}$$

To identify Σ_*h*_, (*I*_*m*_ − *SP*) must be invertible and therefore of full rank. But *SPS* = *S* implies that *S*′*SPS* = *S*′*S*, where *S*′*S* is positive definite, So, *PS* = *I*_*mk*_.

Then, Rank(*I*_*m*_ − *SP*) = *tr*(*I*_*m*_ − *SP*) = *tr*(*I*_*m*_) − *tr*(*I*_*mk*_) = *m* − *mk*, and (*I*_*m*_ − *SP*) is rank deficient; consequently, Σ_*h*_ cannot be identified. The covariance matrix of the h-step ahead reconciled forecast errors ${\tilde{e}}_{n}\left(h\right)=Y_{n+h}-{\tilde{Y}}_{n}\left(h\right)$ is:

$$Var(Y_{n+h}-{\tilde{Y}}_{n}\left(h\right)|I_{n})=SPW_{h}P^{\prime }S^{\prime }$$

For any *P* such that *SPS* = *S*, where $W_{h}=E[{\hat{e}}_{t}\left(h\right){\hat{e}}_{t}^{\prime }\left(h\right)|I_{n}]$ is the variance-covariance matrix of the h-step ahead base forecast errors and is given by Eq (3). The purpose is to find the matrix *P* that minimizes the trace of Var $\left(Y_{n+h}-Y_{n}\left(h\right)|I_{n}\right)$ satisfying *SPS* = *S*, which gives the best (minimum variance) linear unbiased reconciled forecasts, which refer to this as MinT(minimum trace) reconciliation which computed as

$$\begin{array}{c} {\tilde{Y}}_{n}\left(h\right)=S(S^{\prime }W_{h}^{-1}S{)}^{-1}S^{\prime }W_{h}^{-1}{\hat{Y}}_{n}\left(h\right) \end{array}$$

Where *W*_*h*_ is the covariance matrix of the base forecast errors. Although *W*_*h*_ does not suffer from a lack of identification, it is difficult to estimate, especially for *h* > 1. There are several methods to estimate *W*_*h*_ such as [10]:

OLS: *W*_*h*_ = *k*_*h*_*I*, ∀*h*, where *k*_*h*_ > 0 [2]. This method is optimal when the base forecast errors are uncorrelated and equivalent.

WLSv: $W_{h}=k_{h}\;\text{diag}({\hat{W}}_{1}),\forall h$, where *k*_*h*_ > 0 and ${\hat{W}}_{1}=\frac{1}{n}\sum_{t=1}^{n}{\hat{e}}_{t}\left(1\right){\hat{e}}_{t}{\left(1\right)}^{\prime }$.

In this case, MinT is described as a weighted least squares (WLS) estimator applying variance scaling.

WLSs: *W*_*h*_ = *k*_*h*_Λ, ∀*h*, where *k*_*h*_ > 0 and Λ = diag(S1) with 1 being a unit column vector of dimension n.

MinT(Sample): $W_{h}=k_{h}{\hat{W}}_{1},\forall h$, where *k*_*h*_ > 0 the unrestricted sample covariance estimator for h = 1.

MinT(Shrink): $W_{h}=k_{h}{\hat{W}}_{1,D}^{*},\forall h$, where $k_{h}>0,{\hat{W}}_{1,D}^{*}=\lambda_{D}{\hat{W}}_{1,D}+(1-\lambda_{D}){\hat{W}}_{1}$, is a shrinkage estimator with diagonal target, ${\hat{W}}_{1,D}$ is a diagonal matrix comprising the diagonal entries of ${\hat{W}}_{1,}$, and λ_*D*_ is the shrinkage intensity parameter. For more detail, refer to [2, 9, 10].

### 2.2 Forecasting models

Two forecasting models were presented briefly in this subsection for generating forecasts for all levels in the hierarchy to show the influence of different forecasting methods for each hierarchical model: ARIMA and ETS models. They are based on different perspectives on the problem and often, but not always, perform differently, although they share some mathematically equivalent models (Oliveira and Ramos [4]).

#### 2.2.1 ARIMA models

ARIMA models are one of the most popular models for forecasting time series, different types of stochastic seasonal and non-seasonal time series can be represented by these models. The general form of seasonal ARIMA models is:

$$\phi_{p}\left(\beta \right)\Phi_{P}(\beta^{S}){\left(1-\beta \right)}^{d}(1-\beta^{S}{)}^{D}yt=c+\theta_{q}\left(\beta \right)\theta_{Q}(\beta^{S})\varepsilon_{t}$$

Where: *ϕ*_*p*_(*β*) and *θ*_*q*_(*β*) are the regular autoregressive and moving average polynomials of orders p and q, respectively, Φ_*P*_ (*β*^*S*^) and *θ*_*Q*_ (*β*^*S*^) are the seasonal autoregressive and moving average polynomials of orders P and Q, respectively, *S* is the period of seasonality, *D* is the degree of seasonal differencing is the degree of ordinary differencing, *B* is backshift operator; *ε*_*t*_ is a white noise process with zero mean and variance *σ*^2^ [4]. For more detail, refer to [11].

#### 2.2.2 Exponential smoothing

Ord et al. [12] extended the work of Snyder [13] by proposing a class of innovation state-space models which considered as underlying some of the exponential smoothing methods. Hyndman et al. [14] and Taylor [15] extended this to include fifteen exponential smoothing methods [16]. These fifteen methods are discriminated based on the nature of the trend and seasonality component observed [17]. Hyndman et al. [18, 19] described two possible innovations in state space models for each of the fifteen models, resulting in thirty different models, they have developed an automatic forecasting method using these models, a triplet (E, T, S) was used. *E*, *T*, *S* stands for error, trend, and seasonality components, respectively. The general model involves a state vector *x*_*t*_ = (*l*_*t*_, *b*_*t*_, *s*_*t*_, *s*_*t*−1_, …, *s*_*t*−*m*+1_)′ and state-space equations have

$$\begin{array}{cc} y_{t}= & w\left(x_{t-1}\right)+r\left(x_{t-1}\right)\varepsilon_{t}\\ x_{t}= & f\left(x_{t-1}\right)+g\left(x_{t-1}\right)\varepsilon_{t} \end{array}$$

where *ε*_*t*_ is a Gaussian white noise process with mean zero and variance *σ*^2^, *μ*_*t*_ = *w*(*x*_*t*−1_). There are two state-space models: one model with additive errors and the other with multiplicative errors. The model with additive errors has *r*(*x*_*t*−1_) = 1, so

$$\begin{array}{c} y_{t}=\mu_{t}+\varepsilon_{t}. \end{array}$$

The model with multiplicative errors has *r*(*x*_*t*−1_) = *μ*_*t*_, so:

$$y_{t}=\mu_{t}\left(1+\varepsilon_{t}\right).$$

Thus, $\varepsilon_{t}=\frac{y_{t}-\mu_{t}}{\mu_{t}}$ is the relative error for the multiplicative model and any value of *r*(*x*_*t*−1_) will lead to identical point forecast for *y*_*t*_ [17]. For more detail, refer to [14, 18, 19].

### 2.3 Combined forecasts

Combined forecasts were introduced by Bates and Granger [20], and several forecast combination methods have been developed in the literature [21, 22]. In this study, five combination methods were used: The Simple Average method, Geometric Mean, Variance—Covariance method, AKAIKE Weights, and AC method.

#### 2.3.1 Simple Average (SA)

In this method, the forecasts are combined by assigning equal weights to each individual forecast. The combination forecast can be expressed as

$$f_{c}=\sum_{i=1}^{n}w_{i}\;f_{i}$$

Where *f*_*i*_ is the *i*^*th*^ single forecast; *f*_*c*_ is the combined forecast generated by the *n* single forecast *f*_*i*_; *n* is the total number of individual forecasting models, based on Wong et al. [23, 24], and *w*_*i*_ is the combination weight assigned to *f*_*i*_ which is specified as $w_{i}=\frac{1}{n}$.

#### 2.3.2 Geometric Mean (GM)

Suppose the combined forecasts from two forecasting models, the combined forecast using Geometric Mean can be expressed as:

$$f_{c}=\sqrt{\left(f_{1t}\right)\left(f_{2t}\right)}$$

Where *f*_*c*_ is the combined forecast based on the individual forecasts of *f*_1*t*_ and *f*_2*t*_.

#### 2.3.3 Variance-Covariance (VACO)

This method was proposed by Bates and Grager [20]. Due to Shen et al. [25, 26], Suppose the combined forecasts from two unbiased forecasting models are given as

$$f_{c}=wf_{1t}+\left(1-w\right)f_{2t}$$

where *f*_*c*_ is the combined forecast based on the individual forecasts of *f*_1*t*_ and *f*_2*t*_, *w* and (1 − *w*) are the weights assigned to *f*_1*t*_ and *f*_2*t*_, respectively. The weight that minimizes the combined forecast variance is:

$$\begin{array}{c} w=\frac{\sigma_{22}^{2}-\sigma_{12}}{\sigma_{22}^{2}+\sigma_{11}^{2}-2\sigma_{12}} \end{array}$$ (4)

where $\sigma_{11}^{2}$ and $\sigma_{22}^{2}$ are the unconditional individual forecast error variance and *σ*_12_ is the covariance. In practical, Bates and Granger [20] suggested Eq (4) to combine the forecasts

$$\begin{array}{c} w_{i}=\frac{\sum_{t=1}^{T}e_{1t}^{2}}{\sum_{t=1}^{T}e_{1t}^{2}+\sum_{t=1}^{T}e_{2t}^{2}} \end{array}$$ (5)

Where *e*_1*t*_ and *e*_2*t*_ are individual forecast errors, and *T* is the sample size. For more than two individual forecasts the weights can be calculated, according to Fritz et al. [27] by

$$\begin{array}{c} w_{i}=\frac{{\left[\sum_{t=1}^{T}e_{1t}^{2}\right]}^{-1}}{\sum_{i=1}^{n}{\left[\sum_{t=1}^{T}e_{it}^{2}\right]}^{-1}} \end{array}$$

#### 2.3.4 AKAIKE weights

In this method, Akaike’s Information Criterion (AIC) is computed for each model, (Burnham and Anderson [28]; Acquah [29]; Hsiao and Wan [30]; Pi latowska [31]) and the weights can be calculated as follows

$$\begin{array}{cc} w_{i}= & \frac{exp(-0.5\Delta_{i})}{\sum_{j=1}^{N}exp\left(-0.5\Delta_{j}\right)},\;\;\;\;i=1,\cdots ,N\\ \Delta_{i}= & AIC_{i}-AIC_{min} \end{array}$$

Where *AIC*_*min*_ is the minimum of the *N* different *AIC*_*i*_ values.

#### 2.3.5 AC

Altavilla and Ciccarelli [32] Proposed a new methodology that modifies (Aggregated Forecast Through Exponential Reweighting (AFTER) proposed by Yang [33], for more detail, refer to [33, 34]). the weight attributed to a certain model at time *τ* is larger the larger its ability to forecast the actual value not in all previous periods, but only at *τ* − 1.

The weights are assigned as follows:

$$\begin{array}{cc} w_{j,1}= & \frac{1}{j}\\ w_{j,\tau }= & \frac{exp(-\frac{{\left(y_{\tau -1}-{\hat{y}}_{j,\tau -1}\right)}^{2}}{2S_{\tau }^{2}})}{\sum_{j^{\prime }}exp(\frac{{\left(y_{\tau -1}-{\hat{y}}_{j^{\prime },\tau -1}\right)}^{2}}{2S_{\tau }^{2}})},\;\tau \geq 2 \end{array}$$

Where: $S_{\tau }^{2}$ the sample variance of dependent variable, $S_{\tau }^{2}={\left(\tau -1\right)}^{-1}\sum_{s=1}^{\tau -1}{\left(y_{s}-\mu_{\tau }\right)}^{2}$, and $\mu_{\tau }=\tau^{-1}\sum_{s=1}^{\tau -1}y_{s}$

## 3 Data

**Fig 1:**
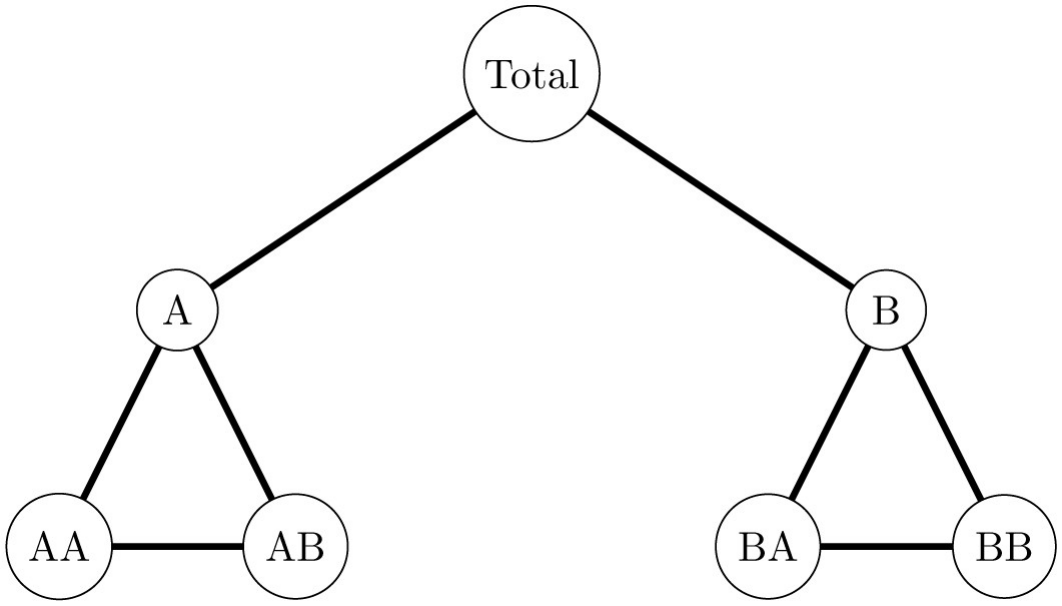
The hierarchical structure of the time series.

The hierarchical structure of the dataset was illustrated in Fig 1, and the number of series at each hierarchical level was presented in Table 1.

**Table 1:** Number of time series per hierarchical level.

Hierarchical level	Number of series
Level 0	1
Level 1	2
Level 2	4
Total	7

**Fig 2:**
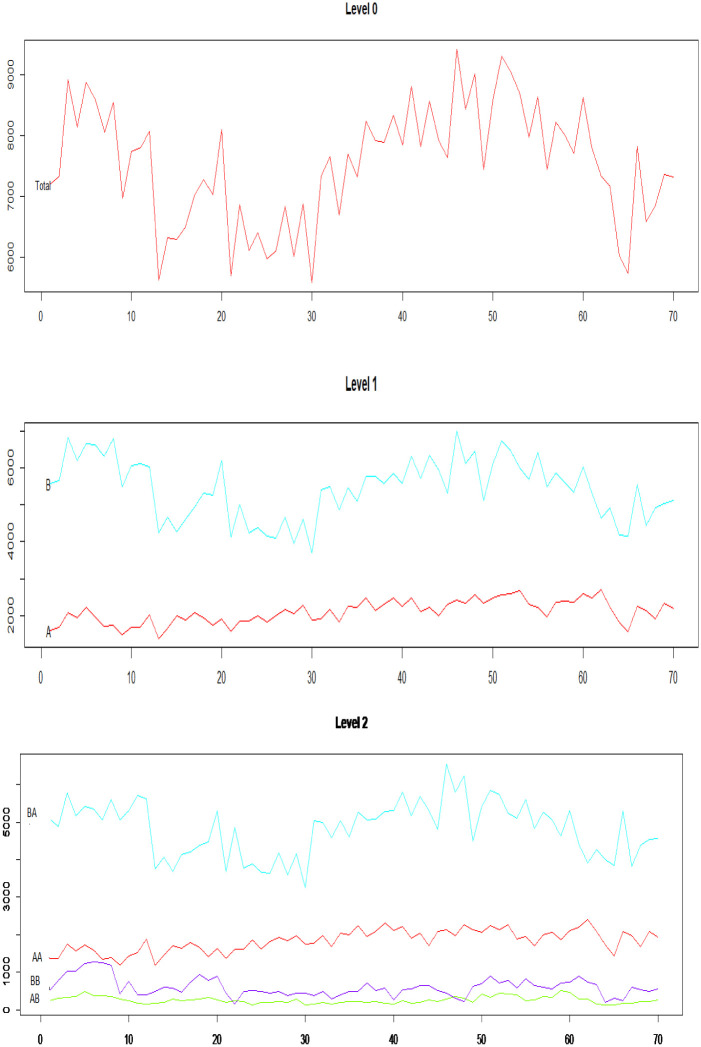
Hierarchical time series.

Table 1 concludes that the hierarchy consists of three levels, the top-level (level 0) represents the international total trade, the middle level (level 1) total exports, and total imports separately, and the bottom level (level 2) shows the way disaggregated. Fig 2 shows the characteristics of the disaggregated series. Thus, the hierarchy includes 7-time series, each containing 70 monthly observations from January 2015 to October 2020 as an estimation period and the data from November 2020 to April 2021 as a testing period, the data were obtained from the Central Agency for Public Mobilization and Statistics (EGYPT). https://www.capmas.gov.eg/Pages/Publications.aspx?page_id=5107&Year=23614.

According to Fig 2, non-Petroleum Imports constitute a larger percentage of the total imports, also non-Petroleum Exports constitute a larger percentage of the total Exports. Generally, there is a Fluctuation as shown in Fig 2.

## 4 The results

This section provides the empirical results obtained, starting with presenting the descriptive statistics of the data, then comparing the performance between hierarchical approaches, and finally comparing the performance of the individual forecasts with the combined forecasts.

### 4.1 Descriptive statistics

The descriptive statistics of the series are presented in Table 2. Which indicates that the mean of the BA (Non-Petroleum Imports) is the highest, the minimum is 121 for Export AB (Crude Oil & its Products), and the data is skewed to the left as indicated by the skewness value except AB and BB (Crude Oil & its Products). Kurtosis values indicated that most of the data are leptokurtic.

**Table 2:** Descriptive statistics value in (MIL) $.

Series	Total	A	B	AA	AB	BA	BB
Mean	7530	2099	5430	1838	261	4826	604
Median	7678	2122	5489	1876	244.5	5038	559.5
Maximum	9413	2694	6989	2406	508	6549	1274
Minimum	5584	1373	3701	1189	121	3253	154
St.dev	970.7692	310.5891	816.6269	290.8765	91.9047	727.1545	246.7991
Skewness	-0.2236	-0.1565	-0.1573	-0.3036	0.7863	-0.1437	0.8569
Kurtosis	2.2733	2.3054	2.1377	2.2268	3.0639	2.2849	3.701

### 4.2 Results from individual models

Mean Absolute Percentage Error (MAPE), and Root Means Error (RMSE) were used as the most popular forecast error measures. MAPE is a relative measure of performance, which specifically was used frequently in the literature studies of hierarchical time series. The MAPE was used in a comparison with the RMSE measure:

$$\begin{array}{cc} & \text{MAPE}=\frac{1}{n}\sum_{t=1}^{n}|\frac{y_{t}-{\hat{y}}_{t}}{y_{t}}|\\ & RMSE=\sqrt{\frac{\sum_{t=1}^{n}(y_{t}-{\hat{y}}_{t}{)}^{2}}{n}} \end{array}$$

Where ${\hat{y}}_{t}\&y_{t}$ are the estimated and actual values respectively, *n* is the number of data (Silveira and Azevedo [6]).

Tables 3–6 contain the values of the MAPE and RMSE for each forecast horizon yielded by bottom-up, top-down, and the optimal combination approach as described in section 2.1, and the last column contains the average across all forecast horizons for ETS and ARIMA models using R software.

**Table 3:** MAPE for out-of-sample forecasting of the hierarchical approaches (ETS).

	Forecast horizon(h)						
	1	2	3	4	5	6	Average
Level 0							
Bottom-up	6.87	12.78	12.56	13.90	16.06	16.42	13.09
Top-down (TDGSA)	6.99	12.89	12.67	14.01	16.16	16.52	13.21
Top-down (TDGSF)	6.99	12.89	12.67	14.01	16.16	16.52	13.21
Top-down (TDFP)	6.99	12.89	12.67	14.01	16.16	16.52	13.21
OLS	6.95	12.85	12.63	13.97	16.13	16.48	13.17
WLS	6.91	12.82	12.60	13.94	16.09	16.45	13.14
MinT (Sample)	5.29	11.30	11.07	12.43	14.63	14.99	11.62
MinT (Shrink)	6.69	12.61	12.39	13.73	15.90	16.25	12.93
Level 1							
Bottom-up	5.03	12.17	12.19	13.69	16.39	16.90	12.73
Top-down (TDGSA)	7.56	14.36	14.34	15.79	18.13	18.79	14.83
Top-down (TDGSF)	7.70	14.47	14.46	15.90	18.42	18.89	14.97
Top-down (TDFP)	5.46	12.54	12.55	14.05	16.72	17.23	13.09
OLS	5.32	12.42	12.44	13.94	16.62	17.13	12.98
WLS	5.21	12.33	12.34	13.84	16.53	17.04	12.88
MinT (Sample)	3.74	10.95	10.97	12.49	15.21	15.73	11.52
MinT (Shink)	5.00	12.13	12.15	13.65	16.34	16.86	12.69
Level 2							
Bottom-up	11.41	18.26	17.58	18.11	20.94	22.30	18.10
Top-down (TDGSA)	15.44	20.18	18.69	19.94	22.20	23.32	19.96
Top-down (TDGSF)	15.59	20.17	18.62	19.92	22.16	23.27	19.96
Top-down (TDFP)	11.37	18.39	17.77	18.32	21.14	22.51	18.25
OLS	12.30	18.94	18.19	18.68	21.41	22.75	18.71
WLS	11.39	18.31	17.66	18.19	21.01	22.39	18.16
MinT (Sample)	13.67	18.54	17.06	18.03	20.45	21.61	18.23
MinT (Shink)	11.91	18.42	17.60	18.17	20.92	22.25	18.21

Where,

Top-down (TDGSA): Top-down forecasts are based on the average historical proportions.

Top-down (TDGSF): Top-down forecasts are based on the proportion of historical averages.

Top-down (TDFP): Top-down forecasts using forecast proportions.

OLS: The function uses ordinary least squares for the unscaled forecasts.

WLS: Weighted least squares for the forecasts scaled by the standard deviation of the forecast errors.

MinT (Sample): Minimum Trace Sample estimator.

MinT (Shrink): Minimum Trace shrinkage estimator.

**Table 4:** MAPE for out-of-sample forecasting of the hierarchical approaches (ARIMA).

	Forecast horizon(h)						
	1	2	3	4	5	6	Average
Level 0							
Bottom-up	3.90	9.80	9.33	10.60	12.72	12.98	9.89
Top-down (TDGSA)	6.94	12.84	12.62	13.96	16.12	16.47	13.16
Top-down (TDGSF)	6.94	12.84	12.62	13.96	16.12	16.47	13.16
Top-down (TDFP)	6.94	12.84	12.62	13.96	16.12	16.47	13.16
OLS	5.81	11.69	11.36	12.65	14.78	15.08	11.90
WLS	4.91	10.78	10.38	11.64	13.75	14.02	10.91
MinT (Sample)	4.12	9.67	9.08	10.48	12.69	13.01	9.84
MinT (Shrink)	4.42	10.19	9.71	10.99	13.12	13.39	10.30
Level 1							
Bottom-up	2.74	9.81	9.62	11.09	13.75	14.20	10.20
Top-down (TDGSA)	7.52	14.31	14.30	15.74	18.27	18.75	14.82
Top-down (TDGSF)	7.65	14.43	14.41	15.86	18.37	18.85	14.93
Top-down (TDFP)	5.80	12.92	13.00	14.54	17.20	17.74	13.53
OLS	4.98	12.04	12.03	13.53	16.16	16.66	12.57
WLS	3.62	10.69	10.58	12.04	14.69	15.14	11.13
MinT (Sample)	3.60	9.91	9.44	10.91	13.61	14.06	10.26
MinT (Shink)	3.20	10.12	9.91	11.37	14.04	14.48	10.52
Level 2							
Bottom-up	11.70	16.31	14.60	15.89	18.46	19.67	16.11
Top-down (TDGSA)	15.43	20.15	18.66	19.91	22.17	23.29	19.94
Top-down (TDGSF)	15.58	20.14	18.58	19.89	22.13	23.24	19.93
Top-down (TDFP)	11.88	17.91	16.94	18.01	20.80	22.21	17.96
OLS	11.99	18.89	18.39	19.10	21.89	23.33	18.93
WLS	11.51	16.62	15.16	16.35	18.99	20.26	16.48
MinT (Sample)	11.43	16.07	14.39	15.80	18.38	19.60	15.95
MinT (Shink)	11.35	16.34	14.82	15.98	18.65	19.91	16.18

**Table 5:** RMSE for out-of-sample forecasting of the hierarchical approaches (ETS).

	Forecast horizon(h)						
	1	2	3	4	5	6	Average
Level 0							
Bottom-up	528.76	1222.99	1149.94	1266.45	1545.12	1553.33	1211.10
Top-down (TDGSA)	537.31	1230.60	1157.78	1274.43	1552.95	1561.29	1219.06
Top-down (TDGSF)	537.31	1230.60	1157.78	1274.43	1552.95	1561.29	1219.06
Top-down (TDFP)	537.31	1230.60	1157.78	1274.43	1552.95	1561.29	1219.06
OLS	534.42	1228.02	1155.13	1271.73	1550.30	1558.60	1216.37
WLS	531.53	1225.45	1152.47	1269.03	1547.65	1555.90	1213.67
MinT(Sample)	406.55	1115.69	1039.02	1153.22	1433.99	1440.32	1098.13
MinT (Shrink)	514.70	1210.50	1137.06	1253.34	1532.25	1540.26	1198.02
Level 1							
Bottom-up	264.38	623.55	583.58	639.10	791.68	792.56	615.81
Top-down (TDGSA)	268.65	616.23	579.97	638.11	785.14	787.83	612.66
Top-down (TDGSF)	268.65	615.94	579.86	638.05	784.77	787.53	612.47
Top-down (TDFP)	268.65	625.04	585.81	614.94	793.79	795.03	613.88
OLS	267.21	624.41	584.96	640.92	792.99	794.12	617.44
WLS	265.76	623.68	584.04	639.84	792.10	793.14	616.43
MinT (Sample)	203.27	567.84	526.69	581.49	734.18	734.46	557.99
MinT (Shrink)	257.35	616.17	576.31	631.98	784.32	785.25	608.56
Level 2							
Bottom-up	183.25	327.62	304.35	337.8	410.51	410.89	329.07
Top-down (TDGSA)	192.47	327.52	306.20	343.84	414.87	414.34	333.21
Top-down (TDGSF)	194.29	327.75	306.53	344.48	415.46	414.76	333.88
Top-down (TDFP)	175.10	328.08	305.21	339.09	411.68	412.20	328.56
OLS	183.25	328.89	305.73	339.35	411.69	412.08	330.17
WLS	179.46	327.56	304.47	338.13	410.79	411.23	328.61
MinT (Sample)	180.83	304.40	280.29	314.83	386.76	385.74	308.81
MinT (Shrink)	181.56	324.75	301.45	335.21	407.73	407.94	326.44

**Table 6:** RMSE for out-of-sample forecasting of the hierarchical approaches (ARIMA).

	Forecast horizon(h)						
	1	2	3	4	5	6	Average
Level 0							
Bottom-up	299.96	1000.57	907.57	1006.13	1274.86	1270.38	959.91
Top-down (TDGSA)	533.53	1227.23	1154.31	1270.90	1549.49	1557.77	1215.54
Top-down (TDGSF)	533.53	1227.23	1154.31	1270.90	1549.49	1557.77	1215.54
Top-down (TDFP)	533.53	1227.23	1154.31	1270.90	1549.49	1557.77	1215.54
OLS	446.94	1138.86	1057.90	1165.58	1438.43	1440.67	1114.73
WLS	377.95	1070.73	983.71	1085.36	1354.72	1352.83	1037.55
MinT (Sample)	317.19	974.60	878.35	994.35	1276.87	1277.26	953.10
MinT (Shrink)	339.94	1022.51	930.95	1034.08	1305.59	1302.81	989.31
Level 1							
Bottom-up	150.41	507.55	459.26	506.79	653.69	648.88	487.76
Top-down (TDGSA)	266.77	614.54	578.23	636.34	783.39	786.06	610.89
Top-down (TDGSF)	266.77	614.30	578.12	636.29	783.02	785.76	610.71
Top-down (TDFP)	266.77	621.39	582.80	639.31	790.25	791.78	615.38
OLS	223.47	575.36	533.57	585.95	733.73	732.53	564.10
WLS	188.98	543.87	498.11	546.95	694.20	690.53	527.11
MinT (Sample)	181.00	498.29	447.36	502.67	655.42	652.81	489.59
MinT (Shrink)	173.36	520.43	472.27	521.65	669.72	665.59	503.84
Level 2							
Bottom-up	148.67	272.50	244.92	277.57	347.13	344.21	272.50
Top-down (TDGSA)	191.68	326.68	305.35	343.00	414.04	413.49	332.37
Top-down (TDGSF)	193.49	326.91	305.681	343.65	414.63	413.93	333.05
Top-down (TDFP)	178.02	328.60	305.54	340.60	412.99	413.24	329.83
OLS	148.68	303.09	278.77	310.92	381.26	381.53	300.71
WLS	158.39	290.50	264.01	296.53	366.46	364.17	290.01
MinT(Sample)	157.20	267.36	238.66	276.21	348.10	345.80	272.22
MinT (Shrink)	154.89	278.31	250.72	283.77	353.82	351.50	278.84

Tables 3–6 concluded that the error percentage produced by the ARIMA model was less than that produced by the ETS model at all levels, and it is clear that the Minimum Trace Sample estimator and the bottom-up approach with ARIMA models have good predictive performance, even with the increased in the forecast horizon. The MinT(shrink) approach is better than optimal combination approaches such as OLS and WLS under the ARIMA model. Moreover, the results obtained from the top-down approach did not present good predictive results.

### 4.3 Results from combining forecasts

To investigate empirically whether combining the forecasts generated from different models can improve forecasting accuracy rather than using individual models is the main focus of this study. So, The forecasts from the best individual hierarchical forecasting approaches were combined (MinT -Sample and the bottom-up) using combination methods as mentioned in section 2.3, and the performance of comparison was evaluated based on the MAPE and RMSE. (See Tables 7 and 8).

**Table 7:** MAPE for combined forecasts.

	Forecast horizon(h)						
	1	2	3	4	5	6	Average
Level 0							
Bottom-up	3.90	9.80	9.33	10.60	12.72	12.98	9.89
MinT (Sample)	4.12	9.67	9.08	10.48	12.69	13.01	9.84
CombSA	4.01	9.74	9.21	10.54	12.71	12.99	9.87
CombGM	4.01	9.74	9.21	10.54	12.71	12.99	9.87
CombVACO	4.02	9.74	9.21	10.54	12.71	12.99	9.87
CombAIC	4.00	9.74	9.22	10.55	12.71	12.99	9.87
Comb(AC)	4.06	9.64	9.06	10.47	12.68	13.00	9.82
Level 1							
Bottom-up	2.74	9.81	9.62	11.09	13.75	14.20	10.20
MinT (Sample)	3.60	9.91	9.44	10.91	13.61	14.06	10.26
CombSA	3.17	9.86	9.53	11.00	13.68	14.13	10.23
CombGM	3.17	9.86	9.53	11.00	13.68	14.13	10.23
CombVACO	3.44	9.86	9.53	11.02	13.70	14.15	10.28
CombAIC	3.11	9.85	9.54	11.02	13.70	14.14	10.23
Comb (AC)	3.25	9.74	9.40	10.89	13.59	14.05	10.15
Level 2							
Bottom-up	11.70	16.31	14.60	15.89	18.46	19.67	16.11
MinT (Sample)	11.43	16.07	14.39	15.80	18.38	19.60	15.95
CombSA	11.57	16.19	14.49	15.85	18.42	19.64	16.03
CombGM	11.56	16.19	14.49	15.85	18.43	19.64	16.03
CombVACO	11.71	16.20	14.51	15.87	18.45	19.67	16.07
CombAIC	11.58	16.21	14.51	15.91	18.48	19.68	16.06
Comb(AC)	11.58	16.11	14.42	15.81	18.40	19.62	15.99

Where,

CombSA: Combined forecasts using Simple average (SA) method.

CombGM: Combined forecasts using Geometric Mean (GM) method.

CombVACO: Combined forecasts using Variance-Covariance (VACO) method.

CombAIC: Combined forecasts using AKAIKE Weights (AIC) method.

Comb(AC): Combined forecasts using (AC) method.

**Table 8:** RMSE for combining forecasts.

	Forecast horizon(h)						
	1	2	3	4	5	6	Average
Level 0							
Bottom-up	299.96	1000.57	907.57	1006.13	1274.86	1270.38	959.91
MinT(Sample)	317.19	974.60	878.35	994.35	1276.87	1277.26	953.10
CombSA	308.57	987.54	892.91	1000.12	1275.76	1273.72	956.44
CombGM	308.58	987.56	892.93	1000.14	1275.78	1273.74	956.46
CombVACO	309.05	987.89	893.14	1000.19	1275.77	1273.66	956.62
CombAIC	307.37	989.35	893.93	1000.95	1275.63	1273.25	956.75
Comb (AC)	312.36	973.82	877.83	994.01	1276.65	1277.09	951.96
Level 1							
Bottom-up	150.42	507.55	459.26	506.79	653.69	648.88	487.77
MinT (Sample)	181.00	498.29	447.36	502.67	655.42	652.81	489.59
CombSA	165.71	502.79	453.30	504.60	654.43	650.71	488.59
CombGM	165.71	502.82	453.23	504.64	654.49	650.78	488.61
CombVACO	172.39	503.10	453.64	505.27	655.14	651.66	490.20
CombAIC	163.58	503.44	454.03	504.89	654.31	650.44	488.45
Comb(AC)	169.93	497.07	447.83	503.34	655.55	652.82	487.76
Level 2							
Bottom-up	148.67	272.50	244.92	277.57	347.09	344.18	272.49
MinT (Sample)	157.20	267.36	238.66	276.21	348.06	345.77	272.21
CombSA	152.94	269.85	241.71	276.80	347.50	344.87	272.28
CombGM	152.92	269.87	241.73	276.83	347.54	344.92	272.30
CombVACO	154.70	270.10	242.05	277.36	348.12	345.66	273.00
CombAIC	152.35	270.21	242.15	281.42	350.81	347.71	274.11
Comb (AC)	154.93	267.52	239.57	277.50	348.95	346.44	272.49

The results of this study showed that the (AC) method works better than other combination methods for forecasting the value of imports and exports, and it is more accurate than the best individual forecasts at level zero (international total trade in Egypt) and level one (total exports and total imports), which leads to providing forecasting values that can be economically relied upon in planning international trade.

## 5 Conclusion

This study used different hierarchical forecasting approaches to obtain the best models and forecasts for international trade in Egypt. ARIMA and Exponential Smoothing (ETS) were used as forecasting models for all levels in the hierarchy. The results concluded that the error rate generated by the ARIMA model was lower than that generated by the ETS model. The minimum trace sample estimator and the bottom-up approaches with ARIMA models have good forecasting performance compared to the other approaches. Furthermore, the forecasts from the Minimum Trace sample estimator and bottom-up approaches with ARIMA models were combined as the best individual approaches using five different combining methods. The results indicated that the (AC) method performs better than other combination methods for forecasting the value of imports and exports, and it is more accurate than the best individual forecasting approaches at level zero (international total trade in Egypt) and level one (total exports and total imports). So, the (AC) method leads to providing forecasting values more accurate than can be economically relied upon in improving the trade balance and drawing up a more efficient production policy. Therefore, the combined forecasts provide a new insight into modeling international trade in Egypt that benefits the government, exporters, and importers. The research recommends using hierarchical forecasting methods in the areas of international trade volume because they produce acceptable forecasts.
